# Evaluating knowledge, awareness, attitudes, and practices regarding vitamin D in pregnant and postnatal Ghanaian women: a cross-sectional study

**DOI:** 10.1186/s12889-025-23986-5

**Published:** 2025-08-02

**Authors:** Benedicta Appiah, Alfred Effah, Samuel Ankomah Danso, Abraham Ameyaw Kwabena, Samuel Kwame Sopuruchi Agomuo, Samuel Kwarteng, Bismark Opoku Mensah, Ebenezer Senu, Enoch Ofori Awuah, Linda Ahenkorah Fondjo

**Affiliations:** 1 Department of Medical Diagnostics, Faculty of Allied Health Sciences, Kwame Nkrumah University of Science and Technology, Kumasi, Ghana; 2 Department of Molecular Medicine, School of Medicine and Dentistry, Kwame Nkrumah University of Science and Technology, Kumasi, Ghana; 3 Department of Biological Sciences, University of Worcester, Worcester, UK; 4 Department of Biological Sciences, School of Natural Sciences and Mathematics, The University of Texas at Dallas, Richardson, TX USA; 5 Kumasi Technical University, Kumasi, Ghana

**Keywords:** Vitamin D, Pregnancy, Awareness, Knowledge, Attitude, Practices

## Abstract

**Background:**

Vitamin D plays a crucial role in maternal and foetal health during pregnancy and lactation. However, its deficiency remains prevalent among pregnant and post-natal women globally, potentially leading to adverse health outcomes. We assessed and compared the knowledge, awareness, attitudes, and practices regarding vitamin D among pregnant and postnatal women in the Greater Accra Region of Ghana.

**Methods:**

This cross-sectional study recruited 310 pregnant women and postnatal women from a Municipal Hospital between January to August 2024. A well-structured questionnaire was used to obtain data on demographic, clinical and lifestyle characteristics of participants. Information on awareness, knowledge, attitude and practices regarding vitamin D were also obtained. The binary logistic regression analysis model was used to determine the independent predictors of awareness and knowledge. *P* < 0.05 was considered statistically significant.

**Results:**

Most pregnant women (82%) were aware of vitamin D, compared to postnatal women (71.1%). Pregnant women also showed higher knowledge levels (62% vs. 50.3%). Nearly all postnatal women (100%) and pregnant women (98.1%) had positive attitudes toward vitamin D, with comparable good practices (88.8% for pregnant, 89.9% for postnatal). Having non-formal education [(aOR = 17.639, 95% CI (3.218–96.686), *p* = 0.001)] or basic education [(aOR = 24.956, 95% CI (6.084-102.366), *p* < 0.001)] was significantly associated with increased odds of non-awareness. Moreover, having basic education [(aOR = 20.946, 95% CI (6.264–70.042), *p* < 0.001)] or SHS education [(aOR = 2.725, 95% CI (1.160–6.402), *p* = 0.021)] were the independent predictors of poor knowledge regarding vitamin D.

**Conclusion:**

Awareness and knowledge of vitamin D were higher among pregnant women compared to postnatal women, with educational level being a significant predictor. Future interventions should prioritize education on the importance of vitamin D, appropriate sun exposure, and dietary sources to mitigate deficiency risks, associated complications, and improve maternal health outcomes during and after pregnancy.

## Introduction

Vitamin D is an immunomodulatory hormone responsible for the regulation of calcium and phosphate metabolism and for maintaining a healthy mineralized skeleton, among other vital body functions [1]. Vitamin D deficiency is a recognized global health challenge, and despite the availability of preventive and treatment options, it remains prevalent [2]. The condition affects individuals of all ages and is estimated to affect one billion people globally [3]. High prevalence of vitamin D deficiency is seen in women of child bearing age, during pregnancy [4] and nursing mothers globally [5]. The deficiency of this hormone is quite common in Ghana, in-spite of the copiousness of sunshine. A prevalence rate of over 30% has been documented across the northern and southern belts of the country, which has been linked to a decrease in vitamin D intake and poor knowledge of the vitamin [6].

Despite the widespread use of prenatal vitamins, vitamin D deficiency is still prevalent across breastfed newborns and pregnant mothers [4, 7]. Children born to vitamin D-deficient mothers have an increased risk of rickets, while maternal vitamin D insufficiency is associated with reduced bone mineral accrual in children [8]. Moreover, research has identified significant associations between low maternal circulating vitamin D levels and preeclampsia [4], altered placental vascular pathology, caesarean section, impaired glucose tolerance, adverse birth outcomes, increased infection rates, as well as altered brain and respiratory function [9, 10]. Pregnancy-related vitamin D supplementation raises the mother’s vitamin D levels and has been linked to better infant growth in the first year of life [11].

Vitamin D is important to maternal health, foetal development, and postnatal life. Poor knowledge and practices regarding vitamin D and its sources have been implicated to contribute to the higher vitamin D deficiency prevalence rates in pregnant and postnatal women. A study from Dublin reported that suboptimal vitamin D levels was common among pregnant women (71%) with insufficient knowledge about vitamin D and its sources [12]. Positive attitude towards sun exposure, diet and supplements can impact lifestyle choices that affect vitamin D intake. Poor practices including limited sun exposure and inadequate dietary intake of vitamin D have also been shown to result in vitamin D deficiency among pregnant and lactating women [13]. Consequently, these habits result in low vitamin D content of breast milk predisposing infants who are exclusively breastfed to vitamin D deficiency and its associated adverse effects [14]. Pregnant and postnatal women are recommended to take sufficient vitamin D supplements to prevent both maternal, congenital and neonatal defects [15]. The Institute of Medicine (IOM) recommends that pregnant and lactating women receive 600 international units (IU) and newborns receive 400 IU of vitamin D daily [16].

Low vitamin D levels during pregnancy and the postpartum period are linked to poor health outcomes for mothers, infants and pregnancy complications such as preeclampsia, preterm birth and low birth weight babies [4]. It is therefore imperative to assess the level of knowledge, awareness, practices and attitudes regarding vitamin D among pregnant and postpartum women in Ghana. Understanding these factors is necessary for developing tailored educational interventions to promote optimal behaviours and improve vitamin D supplementation guidelines and sun exposure recommendations during pregnancy and the postnatal period. Therefore, this study assessed and compared knowledge, awareness, practices and attitudes regarding vitamin D among pregnant and postnatal women in the Greater Accra Region of Ghana.

## Materials and methods

### Study design

This study employed an analytic cross-sectional design to investigate pregnant and post- natal women’s knowledge, attitudes, awareness, and practices regarding vitamin D, between March 2024 and August 2024.

### Study site

This study was conducted at Weija-Gbawe Municipal Hospital, located in the Greater Accra Region of Ghana at coordinates 5.5615 N, 0.29035 W, along the Accra-Winneba route. This hospital was selected as the study site due to its role as a major referral center for maternal and child health services within the Weija-Gbawe municipality, offering comprehensive antenatal and postnatal care. The facility has a capacity of over 100 beds and an antenatal clinic with an annual attendance exceeding 1,000 pregnant women. While other health centers in the municipality provide primary care services, including antenatal care (ANC), the Weija-Gbawe Municipal Hospital has a relatively larger patient volume, and availability of specialized maternal health services not consistently offered at smaller health facilities.

### Study population

This study recruited pregnant and postnatal women aged 18 years and above visiting the Weija-Gbawe Municipal Hospital.

### Inclusion and exclusion criteria

This study involved pregnant of all gestational ages and post-natal women between one week and three months after delivery. However, women who were not pregnant or not within the post-natal period specified were excluded.

### Sample size estimation and sampling technique

The sample size was obtained using the Taro Yamane formula [17], which is suitable for estimating sample sizes in finite populations. The formula is expressed as:$\:n=\frac{\text{N}}{(1+\:{\text{N}(\text{e}}^{2}))}$

Where: n = sample size.

N = population size.

e = margin of error.

The population size (N) was estimated at 1000, based on the annual antenatal and postnatal clinic attendance at Weija-Gbawe Municipal Hospital. A margin of error (e) of 0.05 (5%) was used, assuming a 95% confidence level.

$$\:n=\frac{1000}{(1+\:{1000(0.05}^{2}))}$$

*n* = 285.7.

Therefore, the minimum sample size required was 286. However, a total of 310 participants were recruited to improve statistical power and account for non-response. Participants who met the inclusion criteria were recruited using simple random sampling. The sample included both pregnant and postnatal women. To ensure adequate representation, participants were recruited during separate antenatal and postnatal clinic sessions, which are held on different days. Simple random sampling was employed within each session. Participants were asked to pick a paper card from a container containing cards marked with either “No” or “Yes.” Those who picked a card marked with “Yes” were included in the study, while those who picked “No” were excluded.

### Data collection

A well-structured questionnaire was used to obtain data regarding knowledge, awareness, attitudes and practices of pregnant and postnatal women on vitamin D. The questionnaire was adopted and modified based on previous studies consisting of validated items across a range of dimensions [6, 15, 18]. There were two parts sections to the questionnaire. The respondents’ sociodemographic and clinical data, including age, status (pregnant or postnatal), educational level, monthly income, and occupation, as well as past medical and surgical histories, were the main questions for Part A. Information about lifestyle was also requested which included smoking status, consumption of alcoholic beverages, and frequency of outdoor activities.

The awareness, knowledge, attitudes and practices regarding vitamin D was the main focus of Part B. The participants were asked if they were familiar with the term “vitamin D” and what resources they used to learn about its sources and effects on health. The study also measured participant attitudes towards vitamin D and sun exposure by asking them to rate their agreement with various comments. Additionally, questions focused on daily vitamin D consumption (frequency and types of dietary intake of vitamin D-rich foods), daily sun exposure, and daily use of vitamin D (frequency and dosage of vitamin D supplements).

### Scoring of dependent variables

A single question was used to determine awareness: “Yes” for aware and “No” for unaware. Knowledge was evaluated through 8 questions, scoring up to 29 points, with scores ≥ 14 indicating good knowledge and < 14 indicating poor knowledge. Attitude was assessed using 4 questions on a scale where “Strongly disagree” scored 0, “Disagree” scored 1, “Neutral” scored 2, “Agree” scored 3, and “Strongly agree” scored 4, allowing for a maximum score of 16 points. Scores ≥ 8 indicated a good attitude, while < 8 indicated poor attitude. For practices, 5 questions were scored, with a maximum of 8 points, where scores ≥ 4 indicated good practices and < 4 indicated poor practices. The midpoint/median-based scoring system was utilized in this study to categorize knowledge, attitude, and practices (KAP), which is a robust classification method used in several KAP-related studies regarding vitamin D [19–21].

#### Statistical analysis

Microsoft Excel 2019 and SPSS version 26.0 were used for data entry and analysis, respectively. Categorical variables were presented as frequencies and percentages (%). The Chi-square test was used to compare categorical variables (awareness, knowledge, attitude, and practices) between pregnant and postnatal women to assess differences in proportions. The binary logistic regression analysis model was used to determine the independent predictors of vitamin D awareness and knowledge. Missing data were handled using listwise deletion in analyses. The extent of missing data was minimal and not expected to bias the study findings. *P*-values less than 0.05 were deemed statistically significant.

## Results

### Sociodemographic characteristics of the study participants

Of the 310 participants, 51.9% were pregnant women whilst the remaining 48.1% were postnatal women. Most (30.6%) were aged between 25 and 29 years, and were married (68.7%). Majority (42.6%) had tertiary education and were self-employed (48.7%). Regarding monthly income, 19.7% earned less than GH₵500, 32.3% earned between GH₵500 and GH₵1000, 45.2% earned between GH₵1000 and GH₵5000, and 2.9% earned more than GH₵5000. The religious distribution showed that 75.8% were Christians and 24.2% were Muslims. In terms of Body Mass Index (BMI), 18.1% were classified as normal, 42.9% as overweight, and 39% as obese (Table 1).

**Table 1:** Sociodemographic characteristics of pregnant and postnatal women

Variables	Frequency(*n* = 310)	Percentage (%)
**Status**		
Pregnant woman	161	51.9
Postnatal woman	149	48.1
**Age (years)**		
< 25	72	23.2
25–29	95	30.6
30–35	82	26.5
> 35	61	19.7
**Marital status**		
Single	90	29.0
Married	213	68.7
Widow	5	1.6
Divorced	2	0.6
**Educational level**		
Non formal	21	6.8
Basic education	47	15.2
SHS	110	35.5
Tertiary	132	42.6
**Occupation**		
Unemployed	37	11.9
Student	40	12.9
Self employed	151	48.7
Formally employed	82	26.5
**Monthly income (GH₵)**		
< 500	61	19.7
500–1000	100	32.3
1000–5000	140	45.2
> 5000	9	2.9
**Religion**		
Christian	235	75.8
Muslim	75	24.2
**BMI group**		
Normal	56	18.1
Overweight	133	42.9
Obese	121	39.0

Categorical variables were presented as frequencies and percentages. *SHS *Senior High School, *BMI *Body Mass Index

### Clinical and lifestyle characteristics of pregnant and postnatal women

The majority of participants (89%) self-reported not having any health condition, while 11% had one or more conditions, with hypertension being the most common (37.9%), followed by asthma (27.6%). Approximately, 23% reported bone or muscle diseases. Regarding pregnancy trimesters, 50.3% were in the second trimester, 30.6% in the third, and 19.1% in the first. In terms of gravidity, 32.3% were carrying their second pregnancy, 30% their third, 28.1% their first, and 9.7% their fourth. for the postnatal women, 70.5% were within 1–4 weeks postpartum, and 29.5% were 5–8 weeks postpartum. Smoking was rare, with only 1.3% of participants reporting that they smoked, while 21.4% consumed alcoholic beverages and 78.4% did not (Table 2).

**Table 2:** Clinical and lifestyle characteristics of pregnant and postnatal women

Variable	Frequency (*n* = 310)	Percentages (%)
**Do you have any kind of health condition?**		
No	276	89.0
Yes	34	11.0
**If yes**,** what kind health condition**		
Arthritis	1	3.4
Asthma	8	27.6
Body pains	1	3.4
Diabetes	2	6.9
Hepatitis B	2	6.9
Hypertension	11	37.9
Sickle cell disease	1	3.4
Skin rashes	1	3.4
Ulcer	2	6.9
**Do you have any bone or muscle disease**		
No	240	77.4
Yes	70	22.6
**Trimester**		
Trimester 1	30	19.1
Trimester 2	79	50.3
Trimester 3	48	30.6
**Gravidity**		
Gravida 1	87	28.1
Gravida 2	100	32.3
Gravida 3	93	30.0
Gravida 4	30	9.7
**Week(s) after delivery**		
1–4 weeks	98	70.5
5–12 weeks	41	29.5
**Do you smoke?**		
No	306	98.7
Yes	4	1.3
**Consumption of alcoholic beverages**		
No	243	78.4
Yes	67	21.4

Categorical variables were presented as frequencies and percentages

### Awareness and knowledge responses regarding vitamin d amongst pregnant and postnatal women

The Table 3 outlines the participants’ knowledge and perceptions about vitamin D. Majority, 76.8%, received information about vitamin D, predominantly from health professionals (63.2%), with other sources including schools (25.2%), social media (10%), and newspapers (8.1%). Most participants (74.5%) believed the effects of vitamin D on health are important, and 71.6% recognize its benefits for pregnant and lactating women. A significant number (72.3%) associate vitamin D with bone disease, 55.8% with pregnancy, and smaller proportions link it to conditions like cancer (3.5%) and heart disease (2.9%). While 42.7% think vitamin D reduces caesarean deliveries and infertility, a majority (56.3%) were unsure. Participants commonly believe that vitamin D is obtained from diet (71.6%) and sunlight exposure (63.2%), with fewer considering supplements (33.5%) or exercise (1.6%). Good dietary sources identified include vegetables and fruits (60%), fatty fish (29.4%), and milk (24.8%). Most participants (94.2%) agree that outdoor activities are beneficial for sun exposure, and 71% know that vitamin D status can be checked in a laboratory (Table 3**).**

**Table 3:** Awareness and knowledge response regarding vitamin D amongst pregnant and postnatal women

Variable	Frequency(*n* = 310)	Percentage (%)
**Sources of the information**		
Health professionals	196	63.2
Newspapers	25	8.1
Magazines	10	3.2
On the radio	41	13.2
From relatives/friends	8	2.6
At school	78	25.2
Social media	31	10.0
**Effect of VD on health is important**		
No	2	0.6
Yes	231	74.5
Don’t know	77	24.8
**Vitamin D is good for pregnant and lactating women**		
No	2	0.6
Yes	222	71.6
Don’t know	86	27.7
**According to you**,** Vitamin D is relevant in which of the following health conditions?**		
Bone disease	224	72.3
Cancer	11	3.5
Diabetes	3	1.0
Heart disease	9	2.9
Autism	1	0.3
Infertility	15	4.8
Pregnancy	173	55.8
No health effect	11	3.5
**Vitamin D reduces caesarean delivery and infertility**		
No	3	1.0
Yes	132	42.7
Don’t know	174	56.3
**Where do you think the body gets vitamin D from?**		
Diet	222	71.6
Sunlight exposure	196	63.2
Supplements	104	33.5
Exercise	5	1.6
Water	2	0.6
**Good dietary sources of vitamin D include(s)**		
Vegetables and fruits	186	60.0
Milk	77	24.8
Fatty fish (Salmon, Sardines)	91	29.4
Olive oil	35	11.3
Egg	86	27.7
Chicken	74	23.9
Red meat	27	8.7
Breast milk	50	16.1
Don’t know	51	16.5
**Outdoor games/activities are good for exposure to the sun**		
No	1	0.3
Yes	292	94.2
Don’t know	17	5.5
**Vitamin D status can be checked in the health laboratory**		
No	1	0.3
Yes	220	71.0
Don’t know	89	28.7

Categorical variables were presented as frequencies and percentages. *VD* vitamin D

**Fig. 1:**
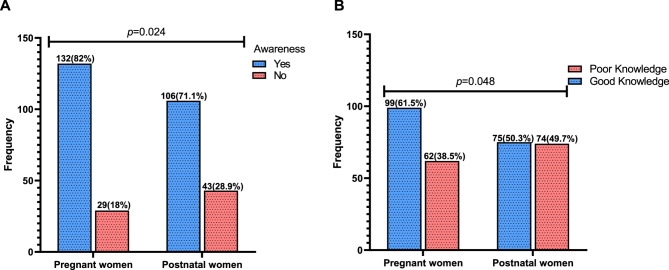
Awareness and knowledge level regarding vitamin D amongst pregnant and postnatal women

Most of the pregnant women (82%) were significantly aware of vitamin D, compared to 71.1% of postnatal women (*p* = 0.024) (Fig. 1A). Similarly, the majority of pregnant women (61.5%) had significantly good knowledge about vitamin D, compared to 50.3% of postnatal women (*p* = 0.048) (Fig. 1B).

### Attitude response regarding vitamin d amongst pregnant and postnatal women

Majority of the participants (55.2%) agree that they like to expose themselves to sunlight to get vitamin D, while 27.7% disagree and 9.7% strongly agree. Regarding sun protection, 46.8% disagree and 31.9% agree that they often use a parasol to shade from the sun, with only 2.6% strongly agreeing. Most participants (72.6%) were willing to undergo a vitamin D test if required by a medical condition. Similarly, 71.6% were willing to take vitamin D supplements (Table 4).

**Table 4:** Attitude response regarding vitamin D amongst pregnant and postnatal women

Variable	Frequency (%)				
	Strongly disagree	Disagree	Neutral	Agree	Strongly agree
**I like to expose to sunlight to get vitamin D**	8(2.6)	86(27.7)	15(4.8)	171(55.2)	30(9.7)
**I often use a parasol (sunshade or umbrella) to shade from the sun**	10(3.2)	145(46.8)	48(15.5)	99(31.9)	8(2.6)
**I am willing to undergo test for vitamin D if a medical condition demands it**	3(1.0)	11(3.5)	24(7.7)	225(72.6)	47(15.2)
**I am always willing to take vitamin D supplements.**	6(1.9)	20(6.5)	46(14.8)	222(71.6)	16(5.2)

Categorical variables were presented as frequencies (%)

**Fig. 2:**
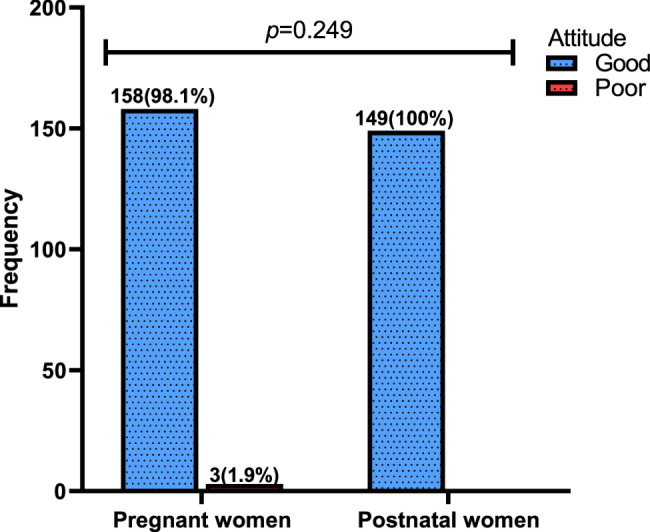
Attitude level regarding vitamin D among pregnant and postnatal women

All the postnatal women (100%) had good attitude towards vitamin D compared to the 98.1% of pregnant women who also demonstrated good attitude levels. The difference in positive attitudes between postnatal and pregnant women was not statistically significant (*p* = 0.249) (Fig. 2).

### Practices response regarding vitamin D amongst pregnant and postnatal women

Majority (60%) did not take vitamin D supplements, while 40% did. Regarding the purchase of vitamin D-rich food, a significant number of participants (87.4%) engaged in this practice. More than half of the participants (64.2%) did not use creams with sun protective factor (SPF). None of the participants (100%) had ever undergone vitamin testing. Considering daily sun exposure, 53.5% spent between 31 and 60 min, 29.7% spent 15 to 30 min, 13.9% spent over 60 min, whiles only 2.9% spent less than 15 min in the sun (Table 5).

**Table 5:** Practices response regarding vitamin D amongst pregnant and postnatal women

Variable	Frequency (%)	
	No	Yes
Taking vitamin D supplements	186(60.0)	124(40.0)
Purchase vitamin D food	39(12.6)	271(87.4)
Not using sun protective factor (SPF) contain creams	111(35.8)	199(64.2)
Ever undergone vitamin testing	310(100.0)	0.0(0.0)
**Average length of time of daily sun exposure**	**Frequency**	**Percentage**
< 15 min	9	2.9
15–30 min	92	29.7
31–60 min	166	53.5
> 60 min	43	13.9

Categorical variables were presented as frequencies and percentages

**Fig. 3:**
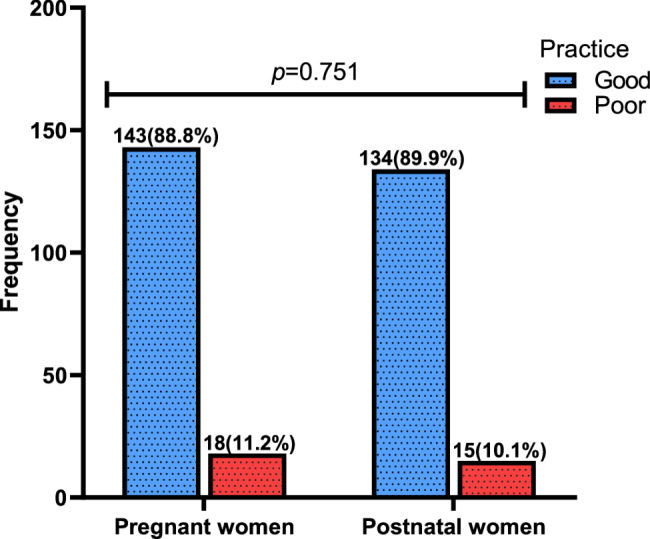
Practices Level Regarding Vitamin D amongst Pregnant and postnatal women

The proportion of good practices regarding vitamin D did not differ significantly between pregnant women (88.8%) and postnatal women (89.9%) (*p* = 0.751) (Fig. 3).

### Sociodemographic and lifestyle predictors of non-awareness of vitamin D among study participants

Following the univariate logistic regression analysis model, being a postnatal woman, educational level, occupation, monthly income, religion, having bone or muscle disease, gravidity and smoking status were the predictors of non-awareness (*p* < 0.05) among study participants.

However, after adjusting for putative confounders in a multivariate logistic regression analysis model, having no formal education [(aOR = 17.639, 95% CI (3.218–96.686), *p* = 0.001)] or basic education [(aOR = 24.956, 95% CI (6.084-102.366), *p* < 0.001)] and smoking (aOR = 21.977, 95% CI (1.348-358.214), *p* = 0.030) were significantly associated with increased odds of non-awareness (Table 6).

**Table 6:** Sociodemographic and lifestyle predictors of non-awareness of vitamin D among study participants

Variables	Non- awareness (*n* = 72)	cOR (95% CI)	*p*-value	aOR (95% CI)	*p*-value
**Status**					
Pregnant woman	29(40.3)	Ref	-	Ref	-
Postnatal woman	43(59.7)	1.846(1.080–3.155)	**0.025**	1.436(0.702–2.935)	0.321
**Marital status**					
Single	25(34.7)	Ref	-	Ref	-
Married	45(62.5)	-	0.211		
Widow	0(0.0)	-	0.999		
Divorced	2(2.8)	-	0.999		
**Educational level**					
Non formal	14(19.4)	31.000(9.765–98.408)	**< 0.001**	17.639(3.218–96.686)	**0.001**
Basic education	31(43.1)	30.031(11.784–76.535)	**< 0.001**	24.956(6.084-102.366)	**< 0.001**
SHS	18(25.0)	3.066(1.277–7.360)	**0.012**	2.224(0.597–8.285)	0.234
Tertiary	8(11.1)	Ref	-	Ref	-
**Occupation**					
Unemployed	19(26.4)	13.370(4.670-38.279)	**< 0.001**	1.566(0.262–9.363)	0.623
Student	5(6.9)	1.810(0.517–6.332)	0.353		
Self employed	42(58.3)	4.881(1.976–12.055)	**0.001**	0.877(0.209–3.677)	0.858
Formal	6(8.3)	Ref	-	Ref	-
**Monthly income**					
< 500	21(29.2)	Ref	-	Ref	-
500–1000	33(45.8)	0.938(0.479–1.839)	0.852		
1000–5000	17(23.6)	0.263(0.127–0.548)	**< 0.001**	1.091(0.251–4.735)	0.907
> 5000	1(1.4)	0.238(0.028–2.034)	0.19		
**Religion**					
Christian	44(61.1)	0.387(0.218–0.685)	**0.001**	0.726(0.336–1.567)	0.414
Muslim	28(38.9)	Ref			
**Bone or muscle disease**					
No	47(65.3)	Ref	-	Ref	-
Yes	25(34.7)	2.281(1.273–4.089)	**0.006**	1.453(0.634–3.330)	0.378
**Gravidity**					
Gravida 1	17(23.6)	Ref	-	Ref	-
Gravida 2	17(23.6)	0.843(0.401–1.774)	0.653		
Gravida 3	25(34.7)	1.514(0.751–3.051)	0.246		
Gravida 4	13(18.1)	3.149(1.286–7.712)	**0.012**	1.456(0.411–5.155)	0.560
**Do you smoke?**					
No	69(95.8)	Ref	-	Ref	-
Yes	3(4.2)	10.304(1.055-100.641)	**0.045**	21.977(1.348-358.214)	**0.030**

Categorical variables were presented as frequencies and percentages; *aOR *Adjusted Odd Ratio, *cOR *crude Odd Ratio, *CI *Confident interval; Binary logistic regression analysis performed to obtain odd ratio; *p*-value<0.05 was considered statistically significant; Bolded *p-*values were statistically significant

### Sociodemographic and lifestyle predictors of knowledge regarding vitamin D among study participants

In a univariate logistic regression analysis model, being a postnatal woman, marital status, educational level, occupation, monthly income, religion and having bone or muscle disease were the predictors of knowledge (*p* < 0.05) among study participants.

However, after adjusting for putative confounders in a multivariate logistic regression model, having basic education [(aOR = 20.946, 95% CI (6.264–70.042), *p* < 0.001)] or SHS education [(aOR = 2.725, 95% CI (1.160–6.402), *p* = 0.021)] were the independent predictors of poor knowledge regarding vitamin D (Table 7).

**Table 7:** Sociodemographic and lifestyle predictors of poor knowledge regarding vitamin D among study participants

Variables	Poor(*n* = 136)	cOR(95%CI)	*p*-value	aOR(95%CI)	*p*-value
**Status**					
Pregnant woman	62(45.6)	Ref	-	Ref	-
Postnatal woman	74(54.4)	1.575(1.003–2.475)	**0.048**	1.163(0.644–2.099)	0.617
**Marital status**					
Single	51(37.5)	Ref	-	Ref	-
Married	82(60.3)	0.479(0.290–0.789)	**0.004**	0.541(0.224–1.305)	0.172
Widow	1(0.7)	0.191(0.021–1.779)	0.146		0.326
Divorced	2(1.5)	-	0.999		0.999
**Educational level**					
Non formal	21(15.4)	-	0.998	-	> 0.999
Basic education	41(30.1)	36.119(13.618–95.799)	**< 0.001**	20.946(6.264–70.042)	**< 0.001**
SHS	52(38.2)	4.915(2.703–8.936)	**< 0.001**	2.725(1.160–6.402)	**0.021**
Tertiary	21(15.4)	Ref	-	Ref	-
**Occupation**					
Unemployed	28(20	18.148(6.886–47.827)	**< 0.001**	2.218(0.509–9.673)	0.289
Student	13(9.6)	2.809(1.140–6.919)	**< 0.025**	0.732(0.176–3.040)	0.667
Self employed	83(61.0)	7.120(3.567–14.213)	**< 0.001**	1.663(0.641–4.313)	0.296
Formal	12(8.8)	Ref	-	Ref	-
**Monthly income**					
< 500	37(27.2)	Ref	-	Ref	-
500–1000	59(43.4)	0.993(0.487–1.788)	0.835	-	-
1000–5000	39(28.7)	0.250(0.133–0.472)	**< 0.001**	1.081(0.290–4.028)	0.907
> 5000	1(0.7)	0.081(0.010–0.690)	**0.021**	0.611(0.49–7.643)	0.702
**Religion**					
Christian	93(68.4)	0.487(0.288–0.826)	**0.008**	1.159(0.548–2.451)	0.715
Muslim	43(31.6)	Ref	-	Ref	-
**Bone or muscle disease**					
No	96(70.6)	Ref	-	Ref	-
Yes	40(29.4)	2.0(1.166–3.429)	**0.012**	1.159(0.548–2.451)	0.699
**Gravidity**					
Gravida 1	40(29.4)	Ref	-	Ref	-
Gravida 2	37(27.2)	0.690(0.384–1.239	0.214	-	-
Gravida 3	40(29.4)	0.887(0.492–1.597)	0.689	-	-
Gravida 4 or more	19(14.0)	2.030(0.864–4.767)	0.104	-	-

Categorical variables were presented as frequencies and percentages; *aOR *Adjusted Odd Ratio, *cOR *crude Odd Ratio, *CI *confident interval; Binary logistic regression analysis performed to obtain odd ratio;* p*-value<0.05 was considered statistically significant; Bolded *p*-values were statistically significant

## Discussion

Although low vitamin D levels during pregnancy and the postpartum period are linked to poor health outcomes for mothers, infants and pregnancy complications such as preeclampsia, preterm birth and low birth weight babies [4], there is a dearth of data regarding pregnant and postpartum women’s knowledge, awareness, practices and attitudes towards vitamin D in Ghana. We assessed pregnant and postnatal women’s knowledge and awareness of vitamin D, as well as their attitudes and strategies for overcoming the stated outcomes. This study showed that pregnant women demonstrated significantly higher awareness and knowledge of vitamin D compared to postnatal women. Educational level significantly influenced awareness and knowledge levels. Notably, participants with lower educational attainment were more likely to have poor knowledge and awareness regarding vitamin D.

We found a notable difference in vitamin D awareness and knowledge, with pregnant women demonstrating higher levels than postnatal women. The study also revealed that health professionals were the primary source of vitamin D information, consistent with findings by Toher and colleagues, emphasizing the crucial role of healthcare providers in disseminating nutritional information before and after pregnancy [12]. Over half of the participants (55.8%) acknowledged the benefit of Vitamin D in pregnancy with 72.3% identifying bone disease as a relevant health condition linked to Vitamin D. This contradicts existing knowledge in Ghana, which showed that the general public, although demonstrating high vitamin D awareness, were unable to identify its specific health benefits and challenges [22]. This demonstrates the effectiveness of educational programs during antenatal care visits in emphasizing the role of Vitamin D in bone health and its broader implications for various health conditions. However, a significant portion of respondents (56.3%) were uncertain about the effect of vitamin D on caesarean delivery and infertility, suggesting a gap in knowledge regarding vitamin D deficiency and pregnancy-related complications. Such findings necessitate the reinforcement of the importance of vitamin D in preventing adverse pregnancy outcomes by healthcare providers.

We found that attitudes towards vitamin D were very positive among both postnatal and pregnant women, and this difference was not statistically significant. This is consistent with an Iranian study that found most pregnant women (91.7%) demonstrating a strong positive attitude towards vitamin D and its importance in maternal and foetal health [23]. Unfortunately, this current research, as well as other studies, has highlighted that a positive attitude does not always translate into optimal practices. Tailored interventions that will equip women with practical strategies offer a solution to bridging the gap between attitude and practice. Additionally, pregnant and postnatal women indicate a generally positive outlook towards sunlight exposure and supplementation, with 55.2% expressing agreement about liking to expose themselves to sunlight for Vitamin D. This is in agreement with Webb et al.’s study, which found that majority of their participants recognized the importance of sunlight for vitamin D synthesis, emphasizing the cultural acceptance of sun exposure as a health-promoting behaviour [24]. The willingness to undergo vitamin D testing (72.6%) and take supplements (71.6%) as expressed by the participants reflects a proactive attitude towards health management, aligning with findings from a study by Rostami et al. which reported high acceptance of vitamin D testing among pregnant women [25]. These positive attitudes highlight the growing awareness of the importance of vitamin D among pregnant and postnatal women, yet they also underscore the need for educational initiatives to enhance understanding of safe sun exposure practices and the importance of regular monitoring of vitamin D levels.

Moreover, our findings showed that both pregnant and postnatal women reported similar good practices regarding vitamin D. A significant majority (87.4%) actively purchase vitamin D-rich foods, suggesting an awareness of dietary sources, which aligns with findings from Wong et al.’s study that highlighted the importance of dietary intake in maintaining sufficient vitamin D levels among pregnant women [26]. Moreover, 64.2% of participants do not use sun protection creams, suggesting an awareness of the importance of UV exposure for vitamin D synthesis and a deliberate choice to avoid blocking it. The average daily sun exposure appears to be adequate for many, with 53.5% spending between 31 and 60 min outdoors, which is generally considered sufficient for vitamin D production [27]. However, 60% of participants do not take vitamin D supplements despite the known benefits of adequate vitamin D levels during pregnancy and lactation. This is particularly concerning, as it contrasts with recommendations from health authorities advocating for vitamin D supplementation in these populations [28]. Again, the complete lack of participants who have undergone vitamin testing indicates a significant gap in health monitoring, suggesting a need for increased awareness and accessibility of vitamin D testing to ensure pregnant and postnatal women are aware of their vitamin D status and subsequently be committed to achieving adequate levels.

This study further revealed various sociodemographic factors that were strongly associated with vitamin D awareness among pregnant and postnatal women. Higher education levels, formal employment, higher income, and Christian religious affiliation were associated with greater vitamin D awareness. These findings align with previous studies, including a study by Parikh et al. which found that higher education and socioeconomic status correlated with better vitamin D awareness among pregnant women [29]. Research indicates that higher vitamin D awareness among women with higher socioeconomic status may be attributed to factors such as a better diet quality, increased health literacy, and more opportunities for outdoor physical activity [30]. The higher awareness among Christians compared to Muslims might be attributed to cultural differences such as clothing choices, which in turn influence sun exposure, vitamin D synthesis, and vitamin D awareness. Women who smoked were also more likely to demonstrate low vitamin D awareness, consistent with studies that found correlations between smoking and vitamin D deficiency [31]. These findings underpin the consideration of socioeconomic barriers, educational disparities, and religious practices in the design of tailored interventions aimed at improving vitamin D awareness and status among pregnant and postpartum women.

Similar to factors influencing vitamin D awareness, lower educational levels emerged as a significant factor significantly associated with poor knowledge levels regarding vitamin D. This is in agreement with a study conducted in India that found that lower educational attainment was linked to inadequate knowledge of vitamin D and its health implications [32]. Educational level likely influences awareness and knowledge through improved access to health information, greater health literacy, and a better ability to understand and apply health-related messages. Individuals with higher education are also more likely to engage with healthcare providers and media sources that promote awareness about micronutrient deficiencies, such as vitamin D, thereby enhancing their knowledge [33, 34]. Educational interventions have therefore been found to increase knowledge, improve practices, and change attitudes regarding vitamin D deficiency in pregnant and postnatal women [35]. Tailored interventions including engagement sessions during antenatal visits, educational outreach programs and community-based awareness campaigns could be employed to improve vitamin D-related awareness and practices. Additionally, married women were more likely to demonstrate poor vitamin D knowledge. Unemployment and lower income levels were also associated with increased likelihood of poor vitamin D knowledge, consistent with findings from a study in Ethiopia that identified socioeconomic status as a significant factor affecting health literacy [36]. These findings collectively necessitate prioritizing pregnant and postnatal women from lower socioeconomic backgrounds to help address barriers, improve vitamin D knowledge, and ultimately reduce adverse maternal and neonatal outcomes.

This study was conducted in a single urban municipal hospital, which may introduce selection bias and limit the generalizability of the findings to the broader Ghanaian population or other regions. Additionally, the cross-sectional design captures data at a single point in time, limiting the ability to establish causal relationships or observe changes over time. The absence of biochemical validation, such as serum vitamin D testing, restricts the ability to correlate reported practices with actual vitamin D status.

## Conclusion

Awareness and knowledge levels regarding vitamin D are higher among pregnant women compared to postnatal women. Educational level was the factor significantly influencing awareness and knowledge levels. Future interventions should focus on educating women about the importance of vitamin D, appropriate sun exposure, and dietary sources to mitigate deficiency risks and associated outcomes during and after pregnancy.

## Data Availability

The datasets used and/or analyzed during this study are available from the corresponding author on request.
